# Telescopic Denture: A Treatment Option for Occlusal Rehabilitation in Partially Edentulous Patients

**DOI:** 10.7759/cureus.35202

**Published:** 2023-02-20

**Authors:** Sorte Nandita Swatantra, Arun Mayya

**Affiliations:** 1 Prosthodontics and Crown and Bridge, A.B. Shetty Memorial Institute of Dental Sciences, NITTE (Deemed to be University), Mangalore, IND; 2 Conservative Dentistry and Endodontics, Manipal College of Dental Sciences, Manipal Academy of Higher Education, Manipal, IND

**Keywords:** removable denture, overdenture, secondary coping, primary coping, telescopic crowns

## Abstract

The rehabilitation of a patient with multiple edentulous spaces, a collapsed occlusal vertical dimension, and compromised abutment teeth is demanding. For treating such patients, various approaches have been put forth over the years, with dental implants having the best results. However, they cannot be used on all patients due to their high cost and surgical limitations. Therefore, removable dentures are still a popular option. Telescopic removable dentures rest on the natural teeth, preserving the alveolar bone while providing good stability and support for the denture. The treatment goal was to establish normal form, function, and aesthetics to uplift the psychological status and maintain occlusal harmony in the patient. In this case report, a 45-year-old female patient who presented with many missing posterior teeth and a collapse in her vertical dimension received full mouth rehabilitation. The patient requested a maxillary denture with no visible metal clasps when smiling. Consequently, a conventional mandibular removable partial denture (RPD) and a cobalt-chromium maxillary telescopic denture were selected as the appropriate treatments. The final restored occlusion and appearance met the patient's satisfaction.

## Introduction

Various treatment modalities have been proposed for partial edentulism, including traditional removable partial dentures (RPDs), teeth/implant fixed partial dentures (FPDs), and teeth/implant overdentures [1]. Due to their numerous benefits, affordability, and ease of fabrication and repair, conventional RPDs have been used frequently. However, metallic clasps used to retain these dentures in place have an unaesthetic appearance and exert lateral forces on the abutment teeth, causing gingival recession and increased wear; this eventually results in bone resorption around the abutment teeth, which reduces the retention and stability of the dentures [2].

An alternative to addressing these issues is telescopic dentures. Abrams and Feder [3], together with Singer and Schön [4], advocated the use of telescopic crowns for removable partial dentures due to their ability to meet the specifications of an abutment retainer. A telescopic crown also functions as a regulating element by minimizing alveolar bone resorption by maintaining the natural abutment teeth [5]. Telescopic crowns are artificial crowns (frameworks) designed to fit over-copings, other crowns, bar connectors, or other suitable rigid supports for dental prostheses. Any removable dental prosthesis that covers and rests on one or more remaining natural teeth, natural tooth roots, or dental implants and a dental prosthesis that covers and is partially supported by natural teeth, natural tooth roots, or dental implants are all examples of telescopic dentures [6].

A primary (inner) coping and a secondary (outer) coping comprise telescopic crowns or double crowns. The abutment teeth are protected from tooth caries and thermal irritants by the primary coping cemented onto them. Additionally, it gives the secondary copings retention and rigidity to engage with the inner coping to form a telescoping unit that acts as an anchor for the remaining teeth [2]. According to the telescopic denture concept, occlusal forces would be transferred from the periodontal ligament of the roots to the alveolar bone. The periodontal ligament's proprioceptive input inhibits occlusal overload, limiting the resorption of the remaining ridge surrounding the roots. Compared to traditional dentures, they offer improved functionality, such as improved biting force, chewing efficiency, and even phonetics. Telescopic dentures are excellent for maintaining oral hygiene and periodontal health since the patient can easily access the abutments [7].

This study describes the treatment of a partially edentulous and periodontally compromised patient. A telescopic prosthesis with chrome-cobalt primary copings, a chrome-cobalt framework as secondary coping for the maxilla, and a conventional cobalt-chromium removable partial denture for the mandible was constructed as full mouth rehabilitation.

## Case presentation

**Figure 1:**
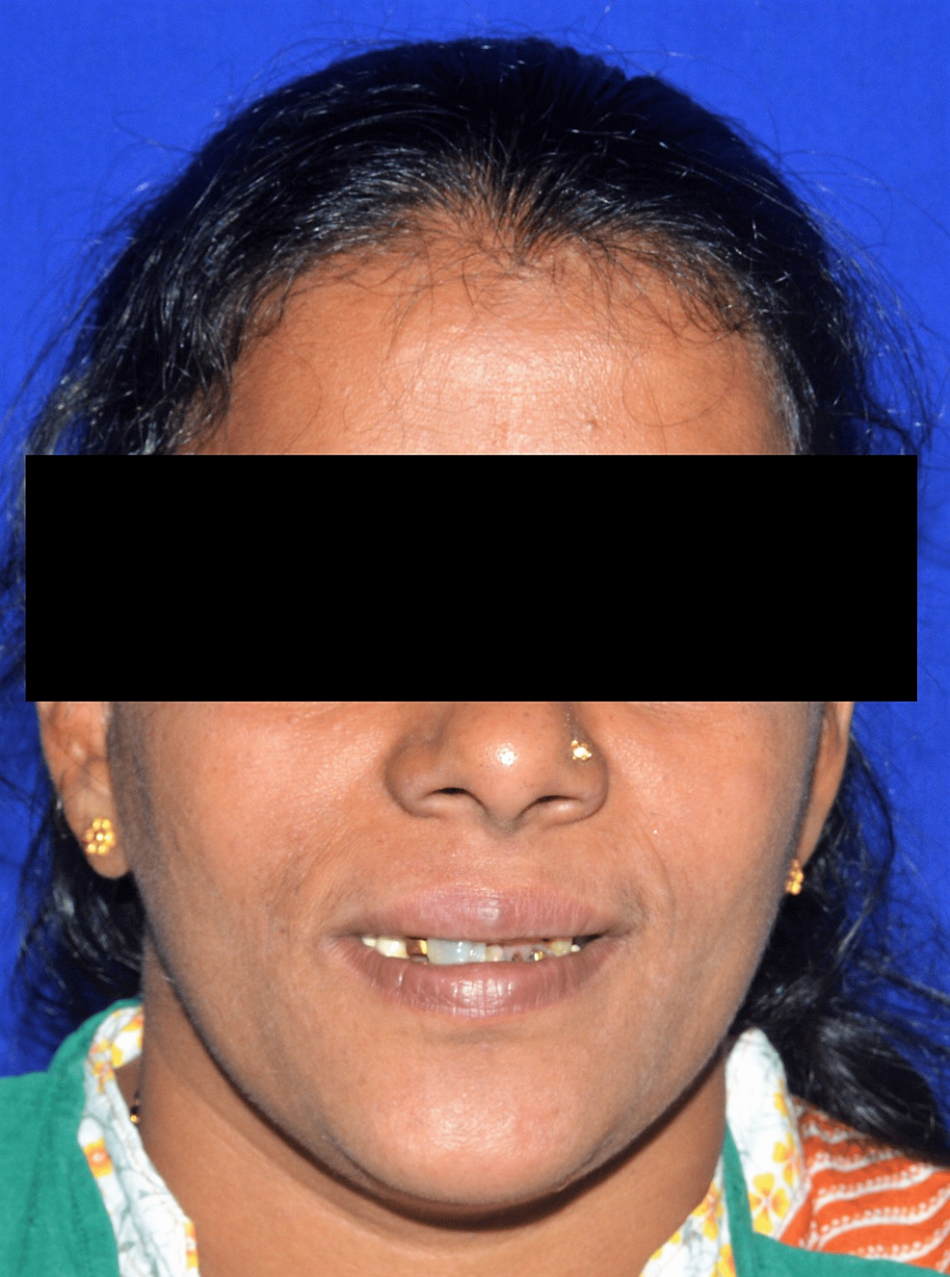
Preoperative extraoral image of the patient

A 45-year-old female patient reported to the Department of Prosthodontics with a chief complaint of missing most of her upper and lower teeth from the past five years (Figure 1).

**Figure 2:**
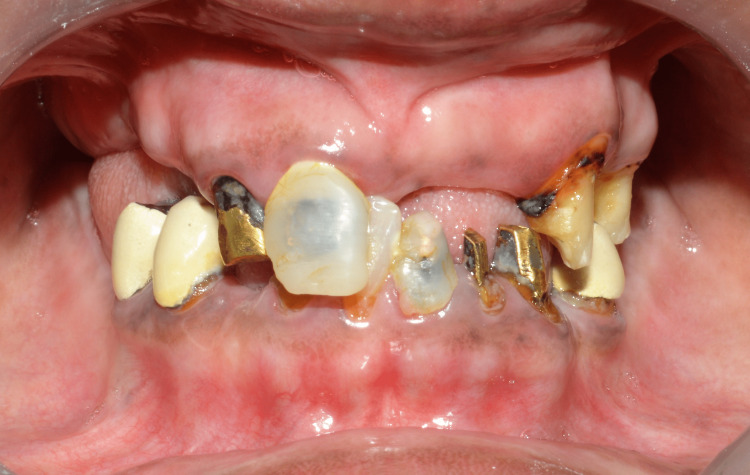
Preoperative intraoral image

**Figure 3:**
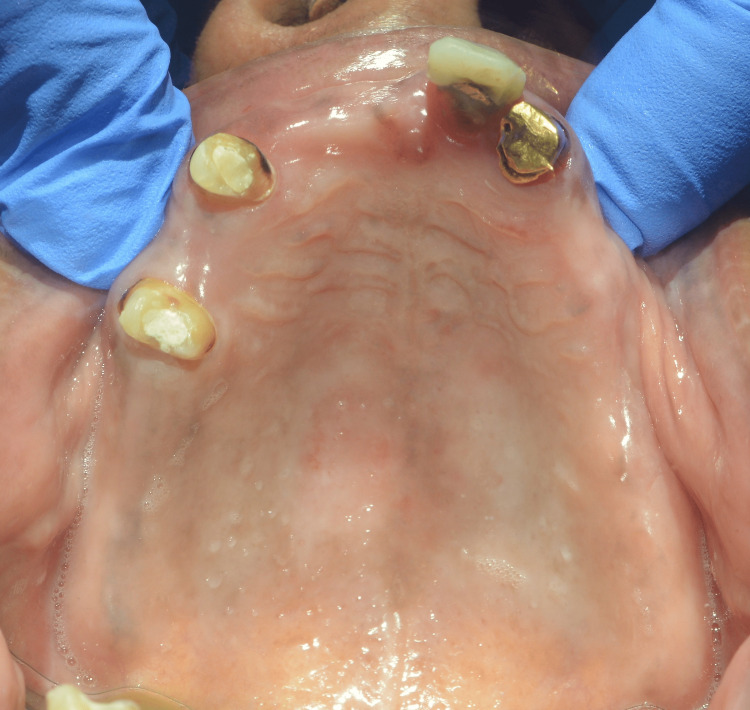
Preoperative intraoral image of the maxillary arch

**Figure 4:**
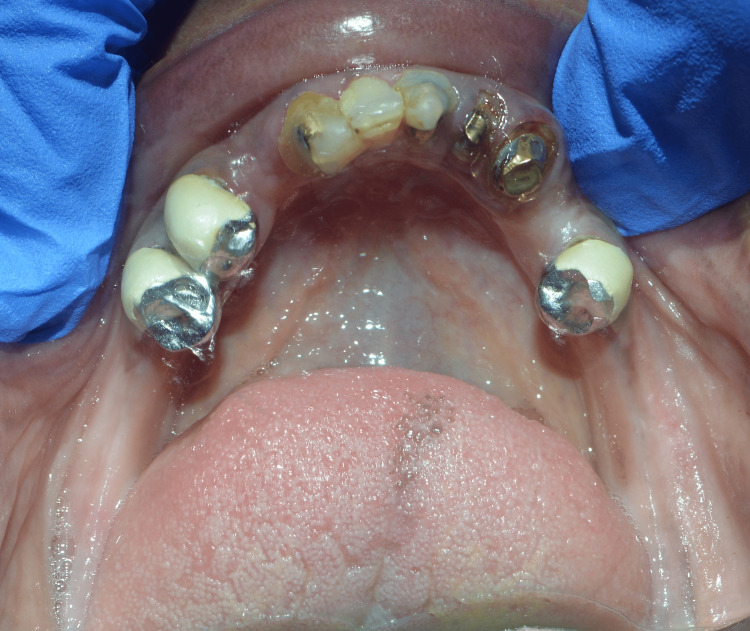
Preoperative intraoral image of the mandibular arch

The patient presented a history of generalized caries and severely decayed teeth, which resulted in the extraction of those teeth. An intraoral examination revealed 11, 12, 23, and 25 and 31, 32, 33, 35, 41, 42, 44, and 45 teeth in the upper and lower arches, respectively, according to the Fédération Dentaire Internationale (FDI) notation system. All teeth were endodontically treated, and post and core was completed in 11, 12, 31, 32, 33, 35, 42, 44, and 45. There were poor, ill-fitting porcelain-fused-to-metal (PFM) prosthetics on the lower premolar teeth. Furthermore, 33, 31, and 11 all had temporary composite crowns. The vertical dimension of occlusion was collapsed (Figures 2-4).

**Figure 5:**
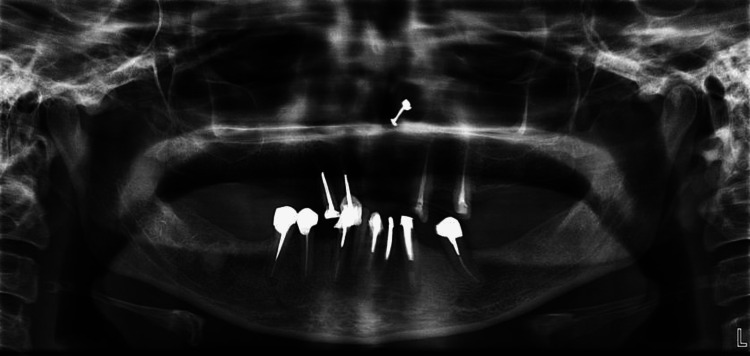
Orthopantomogram (OPG)

All the teeth had good bone support, which radiographic examinations corroborated (Figure 5).

The patient was informed about the various options to rehabilitate her oral cavity, such as implant/tooth-supported removable and fixed dentures. After considering all the factors, it was decided to go ahead with telescopic overdentures for the maxillary arch, PFM crowns for the lower arch, and a cast partial denture for the bilateral distal extension space.

**Figure 6:**
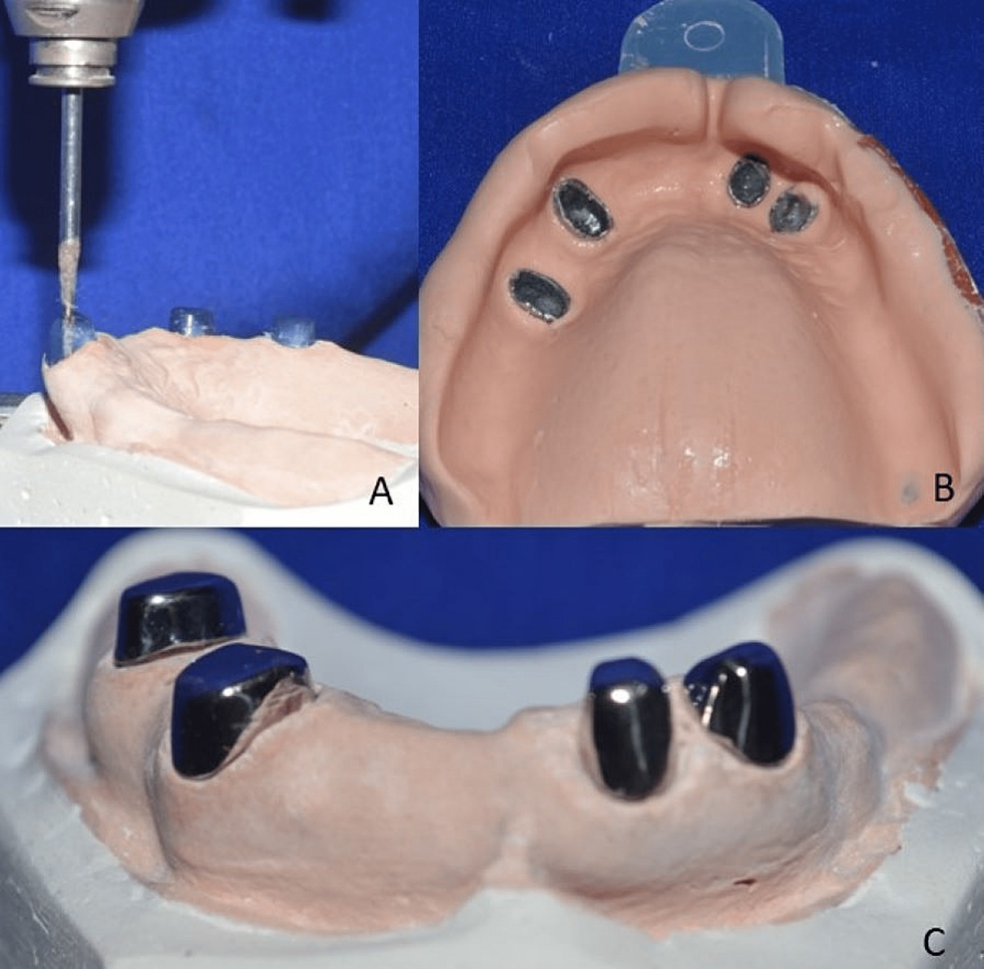
Paralleling of primary copings (A), pickup impression (B), and primary copings on the mastercast (C)

A diagnostic maxillomandibular relationship was recorded to determine the final vertical dimension. In the maxillary arch, teeth preparation with supragingival margins on 11, 12, 23, and 25 was performed to fabricate the primary copings for the telescopic denture. Irreversible hydrocolloid impression material (Algitex, Dental Product of India {DPI}, Mumbai, India) was used to make the impression of the maxillary arch. Cobalt-chromium primary copings with parallel mesial, distal, and labial surfaces were fabricated using the lost-wax technique and were placed on the teeth. A pickup impression was made using alginate, the copings were picked up into the impression, and a mastercast was poured using die stone (Kalabai, Mumbai, India) (Figure 6).

**Figure 7:**
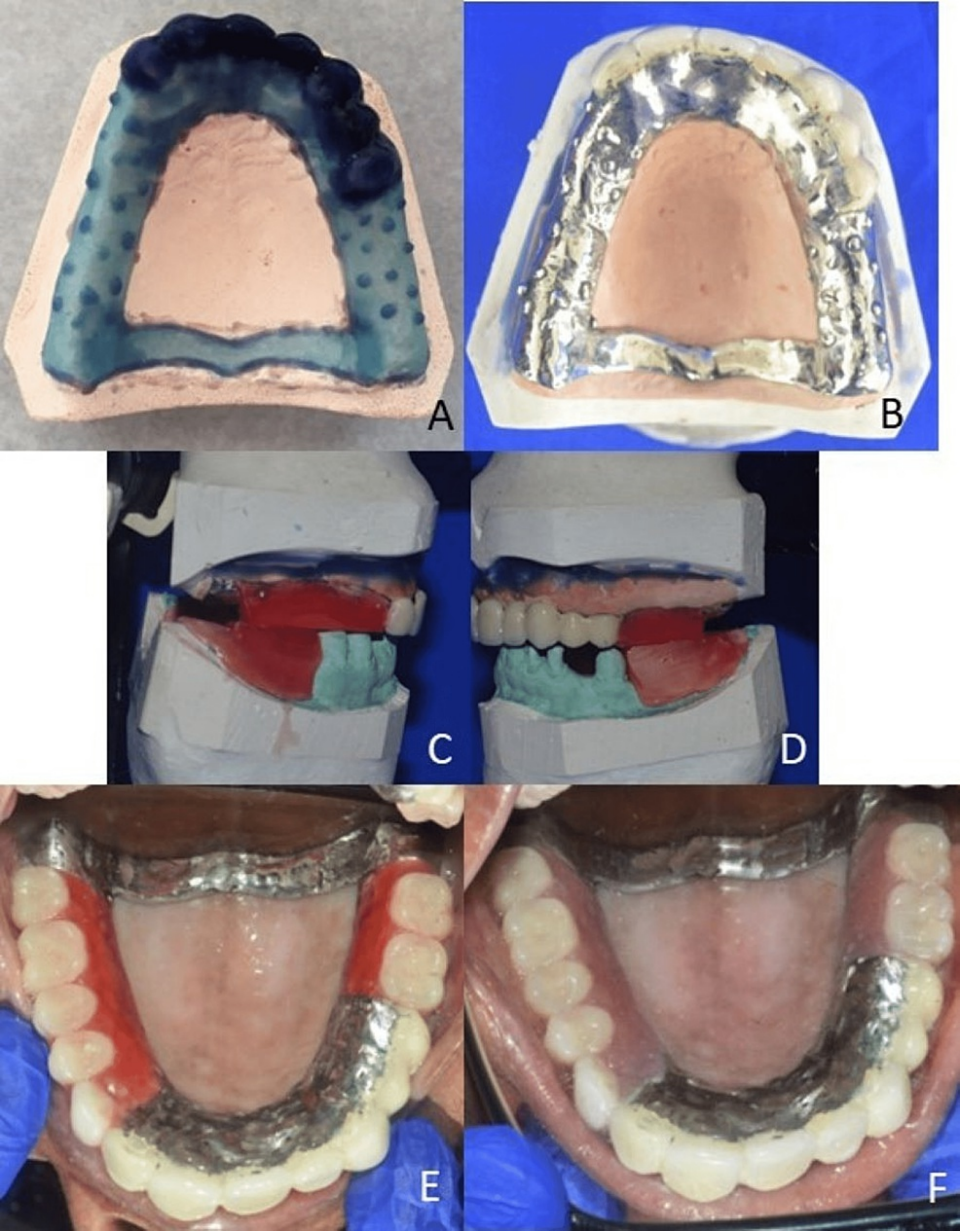
Wax pattern for maxillary telescopic denture (A), telescopic denture after ceramic layering (B), maxillomandibular relationship (C and D), trial with acrylic teeth (E), and final prosthesis (F)

The mastercast was duplicated, and a cobalt-chromium telescopic framework was fabricated and tried intraorally. After that, ceramic was layered on the framework using B2 feldspathic powder (Ceramco 3, Dentsply, Burlington, NJ, USA) and fired in a furnace (Programat P500, Ivoclar Vivadent, Schaan, Liechtenstein). After glazing, the framework was tried in and re-evaluated. After the patient approved of aesthetics, a maxillomandibular relationship was recorded in centric. A trial of the telescopic dentures with acrylic teeth was assessed for occlusion and aesthetics. The maxillary denture was processed using the compression molding technique, and a putty index (Zhermack Zetaplus Putty, Zhermack SpA, Badia Polesine, Italy) was placed around the porcelain teeth to prevent accidental chipping or cracks during acrylization. Final finishing and polishing were done using burs and pumice (Figure 7).

**Figure 8:**
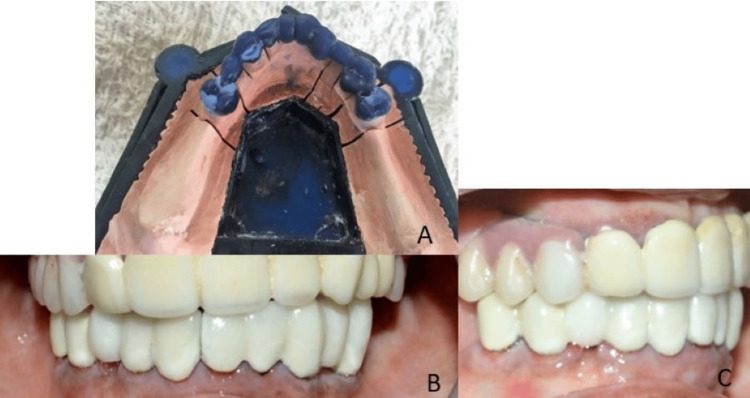
Rest seats carved on wax patterns (A) and crowns cemented (B and C)

In the mandibular arch, teeth preparation for 31, 32, 33, 35, 41, 42, 44, and 45 was done using the diagnostic occlusal rims as a guide for maintaining the vertical dimension. During the wax pattern fabrication of the crowns, the rest seats were carved to receive a cast partial denture as planned. Conventional casting and ceramic buildup procedures were carried out, and the crowns were cemented (Figure 8).

**Figure 9:**
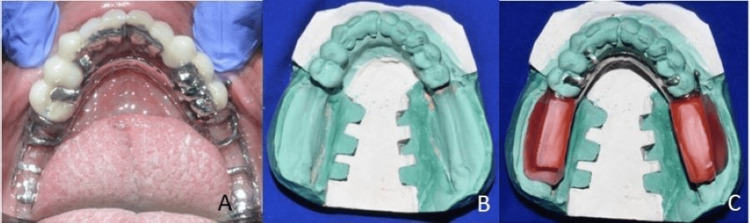
Framework tried intraorally (A) and altered cast technique (B and C)

A cast partial denture was fabricated for Kennedy's class 1 edentulous space using the altered cast technique (Figure 9).

**Figure 10:**
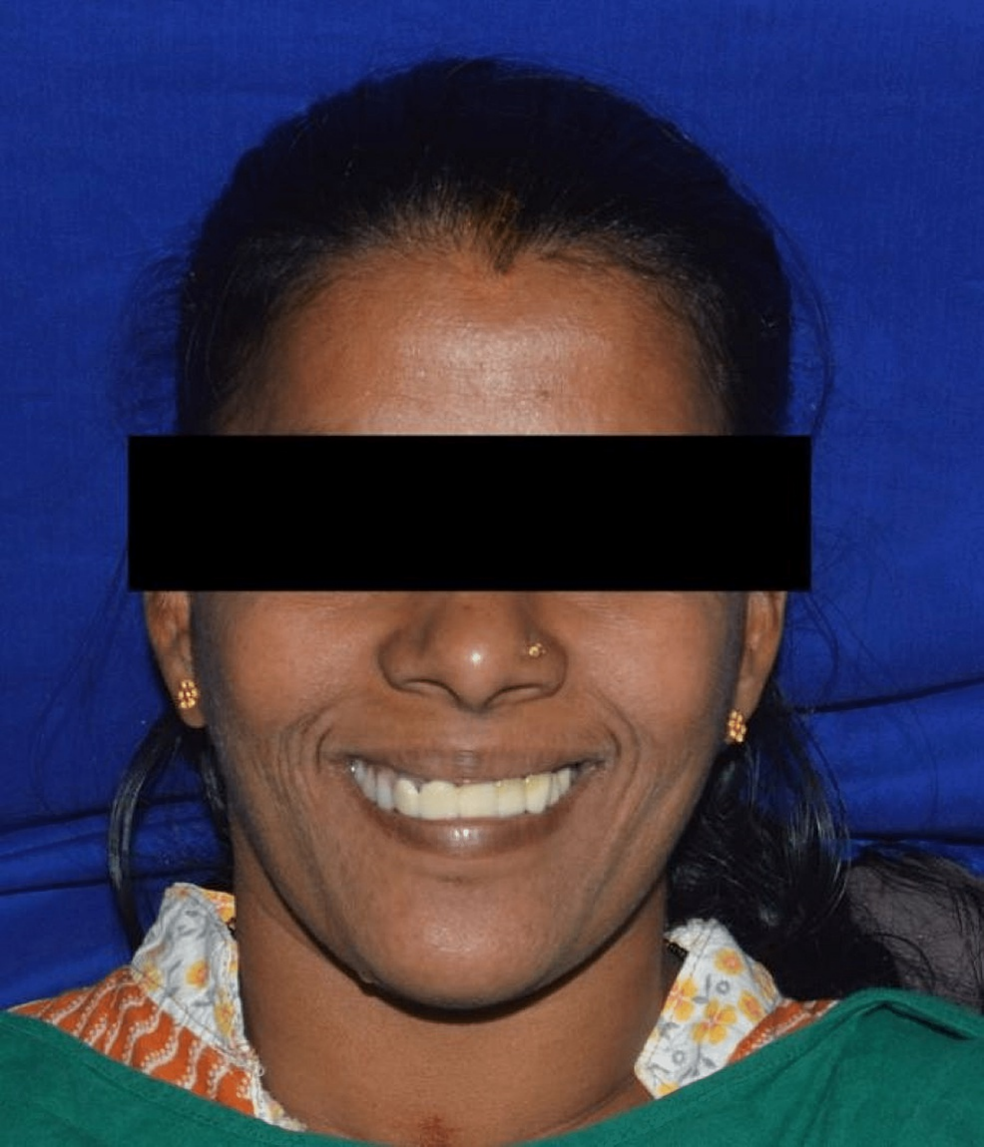
Postoperative extraoral image of the patient

The patient was satisfied with the overall outcome of the treatment (Figure 10).

The patient was assisted in placing and removing the prosthesis. Additionally, the patient received instruction on prosthesis care and hygiene. Following up after six months revealed that the prosthesis had not changed significantly, and the patient was satisfied.

## Discussion

The improvement of a patient's oral structures has a direct impact on their overall quality of life. When structures in the oral cavity are damaged, the ability to chew and taste effectively decreases, which contributes to their state of malnutrition. Therefore, clinicians must help patients regain what they have lost in order for them to maintain a healthy lifestyle.

The prosthetic rehabilitation of patients with multiple edentulous spaces and endodontically compromised abutment teeth has always been challenging [8]. In these situations, the prognosis for fixed prosthodontic therapy is unpredictable. Therefore, traditional dentures, such as removable partial dentures, have been a treatment of choice. However, their effectiveness depends on how much retention and stability the intraoral structures can provide [2]. According to Owall et al., 25% of RPDs were discarded in the first year of patient use due to poor retention and stability. The rapid, progressive alveolar bone resorption that occurs after tooth loss is the reason for the poor retention and stability [9]. Resorption after tooth loss is unavoidable, and the only ways to preserve the remaining bone structure are dental implants or natural tooth roots.

In our case, the upper and lower arches were classified as Kennedy's class I modification 2 (bilaterally distal extension) cases; hence, a fixed dental prosthesis was not considered. A conventional removable denture prosthesis was unfavorable in the upper arch due to a lack of support from the abutment teeth, unlike the lower arch. An implant-supported prosthesis was suggested to the patient, but due to financial constraints and the patient's unwillingness for an invasive procedure, we decided to choose a telescopic prosthesis as the final treatment option.

Telescopic dentures are indicated when a few unevenly distributed teeth are present in the dental arches. Telescopic dentures have better retention and stability due to proprioception, improved stress distribution, and the periodontal ligament's ability to transform compressive forces into tensile forces. There is also a decrease in the rate of residual ridge resorption, which affects the rate of bone remodeling [10]. Dr. J. Beers patented the telescopic crown in 1873 [11], and Langer (1980) improvised them and classified them into three systems: resilient crowns, conical crowns, and cylindrical-shaped inner crowns [12,13]. The cylindrical inner crowns showed excellent retention, but friction between the copings increased wear, and the crowns were challenging to fabricate. Conical-shaped and resilient crowns were well tolerated by the supporting tissues and exerted less stress on the abutment teeth. However, they lacked sufficient retention [1]. According to Schwindling et al., cylindrical crowns outperformed conical and resilient designs with a 90% success rate after seven years [14].

The principal advantages of telescopic dentures are preserving the alveolar bone surrounding the retained teeth and the sensory feedback mechanism from the periodontal fibers, stimulating and guiding dynamic jaw activities [15]. In the case discussed above, the preservation of abutment teeth 11, 12, 23, and 25 maintained the bone support and provided proprioception for the patient; this drastically improved the patient's masticatory function and increased the dentures' stability. The success of a telescopic prosthesis depends on the selection of abutment teeth, the meticulous clinical and radiographic evaluation of the endodontic and periodontal state of the abutment teeth, and the assessment of bone levels and dental health, and also the parallelism maintained in the copings allows the forces to be transferred along the long axis of abutment teeth negating the lateral forces thereby, restricting undesirable tooth movements, maintaining the tooth position, and stabilizing occlusion [15].

According to Ishida et al., a conventional RPD had a 94.5% survival rate, while a telescopic retained RPD had a 100% survival rate. Patients using conventional RPDs frequently complained of greater rates of periodontitis and caries, although there was no statistically significant difference between the two [16].

Frictional surfaces are another aspect that affects retention; materials used for primary and secondary copings should have high shear strengths and resistance to wear. When cobalt-chromium copings were used instead of gold or zirconia inner crowns, retentive forces were found to be higher, according to an in vitro study by Arnold et al. However, other studies have demonstrated that zirconia primary copings have higher long-term retention forces than gold or non-precious metal primary crowns due to their substantially smoother surface under the microscope and great resistance to wear over time [17,18].

## Conclusions

A telescopic denture prosthesis can be a suitable treatment option when there is unfavorable distribution of teeth in the dental arches. However, the treatment planning for these cases is a crucial step in determining the prognosis of the treatment outcome. The dentist has to evaluate the remaining teeth, treat them accordingly, and then plan to use the teeth as abutments. At the same time, due to the technical requirements of the fabrication processes for telescopic dentures, the dental technician also contributes significantly to the success or failure of the prosthesis.

Telescopic prostheses provide superior retention, stability, stable occlusion, and better control of mandibular movement due to the proprioception feedback mechanism, enhancing chewing efficiency and improving phonetics. They are also less harmful to the gingival tissues and have better aesthetics than clasp partial dentures.
